# A New biosensor illuminates the driving force behind mitochondrial outer membrane rupture

**DOI:** 10.1080/27694127.2026.2627062

**Published:** 2026-02-13

**Authors:** Wei-Hua Chu, Wei-Chung Chiang

**Affiliations:** a Program in Molecular Medicine, National Yang Ming Chiao Tung University and Academia Sinica, Taipei, Taiwan; b Institute of Biochemistry and Molecular Biology, College of Life Sciences, National Yang Ming Chiao Tung University, Taipei, Taiwan; c Cancer and Immunology Research Center, National Yang Ming Chiao Tung University, Taipei, Taiwan

**Keywords:** Mitochondrial quality control, PINK1/Parkin-mediated mitophagy, Mitochondrial outer membrane rupture, VCP, Biosensor

## Abstract

In PINK1 (PTEN induced kinase 1)/PRKN (Parkin)-mediated mitophagy, the rupture of the outer mitochondrial membrane (OMM) emerges as a crucial event required for efficient mitochondrial clearance. Mechanistically, OMM rupture exposes inner mitochondrial membrane (IMM) mitophagy receptors, facilitating subsequent autophagic removal. Despite the important role of OMM rupture in mitophagy, the underlying mechanism remains elusive and technically difficult to monitor. In a recent study, we developed a novel fluorescent biosensor to directly visualize OMM rupture. This technique enables temporal and spatial characterization of OMM rupture and provides a powerful platform to dissect the underlying mechanism. Using this tool, we revealed that VCP (valosin containing protein) and its recruitment factors are required for OMM rupture, suggesting that VCP-dependent remodeling of the OMM proteome primes the rupture of OMM during mitophagy.

**Abbreviations**: ARIH1, Ariadne RBR E3 ubiquitin protein Ligase 1; AMFR, autocrine motility factor receptor; ANKRD13A, ankyrin repeat domain-containing protein 13 A; FUNDC1, FUN14 domain containing 1; OA, oligomycin and antimycin; CID, chemical-induced dimerization; IMM, nner mitochondrial membrane; LC3, microtubule-associated protein 1 light chain 3; MUL1, mitochondrial E3 ubiquitin protein ligase 1; NIX, BCL2 interacting protein 3 like; OMM, outer mitochondrial membrane; UBXN1, ubiquitin regulatory X domain-containing protein 1; UBXN6, ubiquitin regulatory X domain-containing protein 6; VCP, valosin-containing protein; WIPI2, WD repeat domain phosphoinositide interacting protein 2.

## Text

Loss of membrane integrity serves as a danger signal that marks damaged compartments to be recognized for subsequent repair or removal by cellular quality control mechanisms. Prior studies have shown that the rupture of endomembranes, including lysosomes, endosomes, and pathogen-containing phagosomes, elicits coordinated cellular responses that recognize membrane damage and, when not repaired, recruit the autophagy machinery for their removal. These observations implied that loss of membrane integrity may represent a more general mechanism of selective organellar autophagy in which inner components of the organelle that get exposed can be sensed by autophagy. Consistent with this notion, the outer mitochondrial membrane (OMM) undergoes PRKN (Parkin)- and proteasome-dependent rupture during PINK1 (PTEN induced kinase 1)/PRKN-dependent mitophagy, allowing topological exposure of inner mitochondrial membrane (IMM) mitophagy receptors that interact with LC3 (microtubule-associated protein 1 light chain 3) proteins and promote autophagic clearance. While these discoveries established OMM rupture as a regulated process that confers substrate selectivity during mitophagy, how OMM becomes ruptured is poorly understood. Thus, we sought to identify the driving mechanisms behind OMM rupture during PINK1/PRKN-dependent mitophagy.

**Figure 1.**
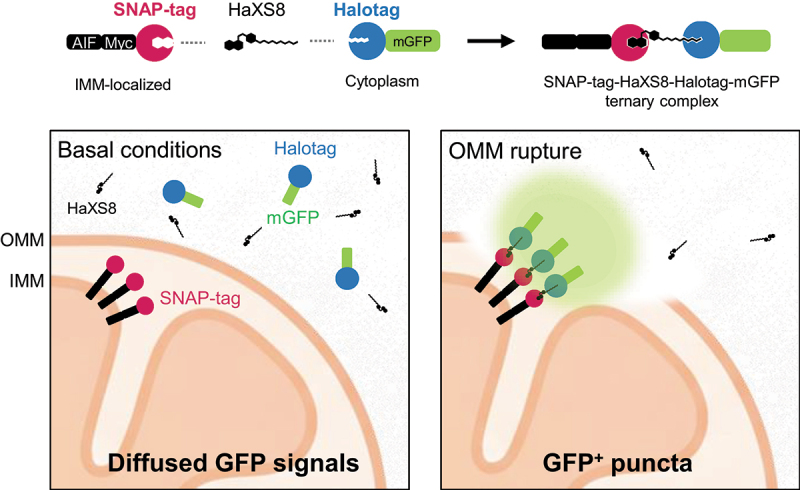
The design of the HaXS8-based OMM rupture sensor. The sensor consists of two components: an IMM-targeted SNAP-tag (AIF-Myc-SNAP-tag) and a cytosolic HaloTag-mGFP probe. Upon OMM rupture, cytosolic HaloTag-mGFP gains access to the IMM, where it covalently conjugates with IMM-anchored AIF-SNAP-tag in the presence of the HaXS8, forming a SNAP-tag–HaXS8–HaloTag-mGFP ternary complex that accumulates into visible mGFP-positive puncta.

Methods commonly used to probe membrane integrity, such as transmission electron microscopy and protease protection assay, are inherently low-throughput and labor-intensive. To facilitate a systematic approach to investigate the molecular mechanism of OMM rupture, we developed an OMM rupture sensor to visualize mitochondria with ruptured OMM^[1]^. The sensor is based on the chemical-induced dimerization (CID) of IMM-targeted SNAP-tag and cytosolic HaloTag-mGFP probes. Upon OMM rupture, the HaloTag-mGFP gains access to the IMM and is covalently conjugated to the SNAP-tag in the presence of HaXS8 dimerizer. The SNAP-tag-HaXS8-HaloTag ternary complex accumulates on the OMM ruptured sites, forming visible fluorescent puncta (Figure 1). This biosensor generates bright mGFP puncta in the majority of HeLa cells expressing PRKN upon the treatment of mitophagy inducers, oligomycin and antimycin (OA), but not in cells under basal conditions. Importantly, the OMM rupture signals were completely abolished in the absence of PRKN or upon pharmacological inhibition of the proteasome, indicating that the biosensor specifically reports the occurrence of OMM rupture during PINK1/PRKN-dependent mitophagy. Further characterization revealed that OMM-ruptured mitochondria are positive for PRKN and overlap with a subset of LC3B- and OPTN (Optineurin)-positive mitochondria, consistent with the model that OMM-ruptured mitochondria are subjected to autophagic sequestration.

With this technique, we observed that the first signs of OMM rupture can appear as early as 2 h post-OA treatment, but they became prevalent from approximately 3 h post-treatment. OMM rupture appears to follow the initial PINK1/PRKN activation and the timing of perinuclear compaction of depolarized mitochondria. However, we cannot exclude the possibility that OMM rupture occurs at an earlier time point, as detection may be limited by the probe sensitivity.

This new tool enabled us to have an unprecedented approach to genetically or pharmacologically identify molecular determinants of OMM rupture. We demonstrated that VCP (valosin-containing protein) is a key driver of OMM rupture. VCP is an AAA-ATPase that functions to remodel protein complexes and membranes through the extraction of single proteins. VCP was previously shown to relocalize to the damaged mitochondria during mitophagy to mediate proteasomal-dependent removal of OMM proteins and promote autophagosome biogenesis. In support of this, the depletion of VCP recruitment factors required for mitophagy, such as UBXN1 (ubiquitin regulatory X domain-containing protein 1), UBXN6 (ubiquitin regulatory X domain-containing protein 6), WIPI2 (WD repeat domain phosphoinositide interacting protein 2), and ANKRD13A (ankyrin repeat domain-containing protein 13 A), significantly attenuated OMM rupture during mitophagy. We propose that the extraction of specific OMM substrates of VCP may directly or indirectly destabilize the membrane, thereby priming the OMM for rupture. To understand how VCP-dependent OMM remodeling promotes membrane rupture, it will be important in future investigations to identify those OMM proteins that have to be removed to induce OMM rupture.

Is OMM rupture a shared feature among different forms of mitophagy, or is it specific to the PINK1/PRKN pathway? In receptor-mediated mitophagy involving FUNDC1 (FUN14 domain containing 1), BNIP3 (BCL2 interacting protein 3), or NIX (BCL2 interacting protein 3-like), which functions largely through ubiquitin-independent mechanisms, OMM rupture may be less likely to occur. In contrast, mitophagy mediated by other ubiquitin E3 ligases, such as ARIH1 (Ariadne RBR E3 ubiquitin protein Ligase 1), AMFR (autocrine motility factor receptor), MUL1 (mitochondrial E3 ubiquitin protein ligase 1), involves OMM ubiquitylation, potentially triggering similar proteasome/VCP-dependent membrane destabilization. With our new OMM rupture sensor, these possibilities can now be formally evaluated.

OMM rupture may facilitate the release of pro-apoptotic factors, thereby promoting unconventional apoptotic cell death. Thus, failure of autophagic removal or excessive accumulation of OMM-ruptured mitochondria following the activation of PINK1/PRKN-dependent mitophagy could be deleterious. This raised an interesting question: What is the physiological consequence of OMM ruptured mitochondria not being properly removed? As defects in autophagy are a common theme in many neurodegenerative and inflammatory diseases, excessive, unmitigated OMM rupture during mitophagy may contribute to disease pathogenesis.

Taken together, the development of the HaXS8-based OMM rupture sensor provides an unprecedented opportunity to directly probe OMM rupture during mitophagy *in cellulo* and dissect the underlying mechanism. Future studies employing this tool will help to expand our understanding of OMM rupture in pathophysiologically relevant contexts and investigate whether similar membrane disruption occurs in other forms of selective organellar autophagy.

## Data Availability

Data sharing is not applicable to this article as no data were created or analyzed in this study.
